# HE4 as a Prognostic Biomarker of Major Adverse Cardiovascular Events in Patients with Abdominal Aortic Aneurysm: A Canadian Prospective Observational Study

**DOI:** 10.3390/biomedicines13071562

**Published:** 2025-06-26

**Authors:** Hamzah Khan, Abdelrahman Zamzam, Farah Shaikh, Muhammad Mamdani, Gustavo Saposnik, Mohammad Qadura

**Affiliations:** 1 Division of Vascular Surgery, St. Michael’s Hospital, Toronto, ON M5B 1W8, Canada; hamzah.khan@mail.utoronto.ca (H.K.);; 2 Li Ka Shing Knowledge Institute, Unity Health Toronto, St. Michael’s Hospital, Toronto, ON M5B 1W8, Canadagustavo.saposnik@unityhealth.to (G.S.); 3 Division of Neurology, Department of Medicine, University of Toronto, Toronto, ON M5T 1P5, Canada; 4 Department of Surgery, University of Toronto, Toronto, ON M5T 1P5, Canada

**Keywords:** abdominal aortic aneurysm, major adverse cardiovascular events (MACEs), prospective observational study, Canada

## Abstract

**Background:** Abdominal aortic aneurysm (AAA) is a chronic inflammatory disease characterized by the proteolytic breakdown of the extracellular matrix. A clinical biomarker is needed for risk stratification and prognosis. **Methods**: In this single-center, 5-year observational study, 452 patients were enrolled: 343 with AAA (≥3 cm), and 109 controls (<3 cm). Plasma levels of six inflammatory proteins (human epididymis protein 4 (HE4), matrix metalloproteinase (MMP) 1 and 3, cathepsin S, chitinase 3 like-1, cathepsin S, and B-cell activating factor (BAFF)) were quantified at baseline. Patients were followed for a total of 5 years (60 months), and major adverse cardiovascular events (MACEs, defined as the composite of myocardial infarction, cerebrovascular attack, and cardiovascular-related death) were recorded. A Cox proportional hazard model was created using biomarker levels, age, sex, hypertension, hypercholesterolemia, diabetes mellitus, smoking status, and coronary artery disease to determine whether the baseline levels of these proteins were associated with MACEs over 5 years. **Results**: HE4, MMP-3, BAFF, and cathepsin S levels were significantly elevated in AAA patients compared to controls (all *p* < 0.05). HE4/WFDC2, MMP-3, and Chitinase 3-like 1 were significantly linearly associated with AAA diameter at baseline. With every normalized unit increase in HE4/WFDC2, MMP-3, and Chitinase 3-like 1, there was an increase in abdominal aortic diameter by 0.154 (95% CI: 0.032–0.276, *p* = 0.013), 0.186 (95% CI: 0.064–0.309, *p* = 0.003), and 0.231 (0.110–0.353, *p* < 0.001) centimeters, respectively. Among patients with AAA, elevated HE4 was associated with higher risk of MACEs (adjusted HR 1.249; 95% CI: 1.057–1.476; *p* = 0.009). Patients with high baseline HE4 (≥9.338 ng/mL) had significantly lower freedom from MACEs at 5 years (76.7% vs. 84.8%, *p* = 0.022). Conclusions: HE4 may be a potential prognostic biomarker that can be used to risk stratify patients with AAA to better personalize treatment strategies to reduce adverse events.

## 1. Introduction

Abdominal aortic aneurysm (AAA), occurring within the suprarenal or infrarenal aorta, is a chronic disease caused by a permanent local dilatation of the artery [1]. This disease affects between 4 and 8 percent of the population over the age of 65, with evidence of increasing prevalence due to aging populations [2]. Despite the increasing advances in treatment options, the risk of cardiovascular complications remains high, estimated around 20–30% [3]. Mechanistically, AAA is characterized by the failure of structural proteins, such as collagen and elastin within the extracellular matrix (ECM), leading to weakening and ballooning of the arterial wall [4]. This weakening can lead to arterial wall rupture and potentially fatal bleeding, with a mortality of over 80% [5]. The cause of the failure of these proteins is not well characterized; however, a hyperinflammatory environment has been hypothesized to influence the ECM and lead to a dysregulation of these proteins and AAA formation [6,7]. A hyperinflammatory environment associated with AAA also puts patients with AAA at an increased risk of major adverse cardiovascular events (MACEs) [3,8,9].

Previous research has demonstrated that patients with AAA have an elevated risk of myocardial infections (MIs), cerebrovascular attacks (CVAs), and all-cause mortality when compared to healthy controls. Despite surgical intervention to treat the disease, some patients remain at an increased risk of these long-term outcomes, sometimes with a higher risk of MACEs [10]. Hence, early detection of patients with an elevated risk of MACEs at baseline is necessary to streamline treatment and surgical planning. A recent literature review of all current prognostic biomarkers for AAA has demonstrated there is no available biomarker that predicts MACEs that is specific for patients with stable AAA [11]. There is a dire need for clinically relevant and accurate biomarkers that can determine patients at risk of MACE, as AAA can be a heterogeneous disease, making it difficult to determine the prognosis and clinical course [12]. This is critically important when deciding between open surgical repair versus endovascular aortic repair (EVAR). In patients with significant comorbidities or higher postoperative risk, EVAR is the preferred surgical treatment, if the aortic anatomy permits, due to its minimally invasive strategy [13]. Having a strong prognostic biomarker will allow physicians to provide patients with tailored treatment plans and make more informed surgical decisions, preventing adverse outcomes earlier.

Due to the inflammatory nature of AAA, there has been a keen interest in discovering novel inflammatory biomarkers for the prognosis of this disease. Human epididymis protein 4 (HE4), also commonly referred to as whey acidic protein four-disulfide core domain 2 (WFDC2) is a protein that was originally discovered within the epididymis, but it is commonly found throughout the lungs and reproductive organs [14]. HE4 has been extensively studied in its role as a biomarker for ovarian cancer [15]. It is function is not fully understood; however, it has a conserved motif found within protease inhibitors and is known to support the innate immune system within certain epithelial membranes [16]. It has been demonstrated to interact with matrix metalloproteinase-9 (MMP-9), which itself has been demonstrated to be a prognostic marker of abdominal aortic events [11]. MMP-1 and 3 have similar functions to MMP-9, and hence, these two additional proteins were included in our analysis. Similarly, Cathespin-S and Chitinase 3-like 1 have been implicated in vascular tissue and ECM degradation and remodeling, a hallmark of aortic aneurysmal disease [17,18]. Lastly, inflammation is a well-known contributor to the progression of aortic aneurysm, as well as cardiovascular disease. B-cell activating factor (BAFF/BLyS) is a marker of inflammation and functions through its ability to increase B-cell proliferation and survival [19]. It has previously been noted that there are elevated numbers of B-cells within AAA lesions [20]. None of these biomarkers have previously been investigated for their association with MACE in patients with stable AAA.

Given that there are 20,000 new AAA diagnoses in Canada and that AAA has been demonstrated to be associated with a marked increase in the risk of cardiovascular events and mortality, there is a strong epidemiological rationale for identifying high-risk AAA patients [21,22]. Despite advances in imaging and surgical techniques, epidemiological studies consistently demonstrate that patients with AAA remain under-managed for their increased risk of MACEs, even in stable disease [23]. The lack of clinically accepted prognostic tools for the prediction of MACEs using protein plasma biomarkers limits targeted intervention strategies in this patient population. This study builds upon epidemiological evidence that highlights the cardiovascular vulnerability of patients with AAA. Our primary objectives for this single-center prospective 5-year pilot study was to determine the association between the six plasma biomarkers of interest and MACEs. The second objective was to risk-stratify patients based on these biomarkers levels to determine 5-year freedom from MACEs.

## 2. Materials and Methods

All protocols were approved by the Unity Health Research Ethics Board (REB #20-195 and #16-375) and all participants in this research study provided written informed consent. All methodology followed the World Medical Association Declaration of Helsinki [24]. This study was reported in accordance with the STROBE (Strengthening the Reporting of Observational Studies in Epidemiology) guidelines [25]

### 2.1. Patient Recruitment

Consecutive patients were prospectively recruited from the St. Michaels Hospital vascular surgery ambulatory clinic in Toronto, Canada, between August 2017 and January 2019 and were followed up after their baseline every 6 months or 1 year (based on SVS guidelines for AAA management) for a total of 5 years [13]. An ultrasound, computed tomography angiography, or magnetic resonance angiography imaging of the aorta, as well as an examination by a certified vascular surgeon, was conducted at each follow-up. Patients were included in the study within the AAA study group if they had an AAA measuring ≥3 cm on imaging and were older than 18 years of age. Selection bias was minimized by prospectively recruiting consecutive patients from a single tertiary vascular surgery center who met strict eligibility criteria. Patients were included in the study within the control group if they did not have a diagnosed AAA, with an abdominal aorta measuring <3 cm on imaging, and were older than 18 years of age. Patients with previous aortic repair, thoracic or thoracoabdominal aortic aneurysms, aortic dissections, mycotic or inflammatory AAA, chronic kidney disease, or receiving current treatment for cancer were excluded.

### 2.2. Baseline Clinical Characteristics

Clinical information was collected at the baseline visit, including abdominal aortic diameter, age, sex, history of hypertension (patients taking blood pressure lowering medication, with systolic blood pressure > 130 mmHg, and diastolic blood pressure > 80 mmHg), hypercholesterolemia (patients taking lipid-lowering therapy with triglyceride levels > 1.7 mmol/L or total cholesterol > 5.2 mmol/L), diabetes mellitus (patients with hbA1c > 6.5%), coronary artery disease and congestive heart failure (defined as a diagnoses documented within clinical chart by cardiologist), smoking status (current, past, or never) [26], and current medication.

### 2.3. Plasma Protein Biomarker Analysis

Blood was collected from the antecubital vein into 3.2% citrated vacutainer tubes on the day of recruitment, and plasma was collected by centrifugation at 1000× *g* for 10 min within 1 h of collection. Plasma samples were stored at −80 degree Celsius until analysis. Biomarker analysis was conducted by thawing the samples on ice and quantifying markers by LUMINEX assay (Bio-Techne, Minneapolis, MN, USA) as described by the manufacturer. Human epididymis protein 4 (HE4), matrix metalloproteinase (MMP) 1 and 3, cathepsin S, B-cell activating factor (BAFF), and chitinase 3 like-1 were quantified in the plasma samples. All samples were run in a single duplicate.

### 2.4. Patient Follow-Up and Outcomes

All patients were followed up at 6 or 12 months after baseline as per the guidelines provided by the Society for Vascular Surgery. At each visit, new imaging was obtained, as well as a physical assessment by a vascular surgeon. Over the 5-year period, MACEs were recorded and included MI, CVA, and cardiovascular-related death (death due to MI, CVA, or aortic rupture). Patients were contacted by telephone at the end of the 5-year period for final data collection (June 2024).

### 2.5. Statistical Analysis

In this pilot, observational, prospective study, continuous variables were presented as mean and standard deviation (mean ± SD) and categorical variables were presented as sample number and percent (*n*(%)). Differences between groups for continuous variables were determined by an independent t test if they were normally distributed and by the Mann–Whitney U test if they were non-normally distributed. For categorical variables, difference between proportions were determined by the chi squared test. Biomarkers were z-score normalized for analysis and summarized as mean ± standard deviation (SD).

For this study, the primary objective was to determine the association between baseline biomarker levels and MACEs over a period of 5 years. The secondary objectives aimed to support the association between biomarker levels at baseline, AAA, and MACEs and included (1) determining whether specific markers were elevated in patients with AAA versus controls and those who have suffered from a MACE versus those who did not; (2) determining the association between inflammatory biomarkers at baseline and aneurysm diameter at baseline; and (3) to determine whether there was a significant difference in freedom from MACEs when risk-stratifying into high or low biomarker level.

To address the primary objective, univariate and multivariate Cox proportional hazard models were used to calculate hazard ratios (HRs) for risk of MACEs with each normalized unit increase in plasma biomarker and reported as HR and 95% confidence interval (95% CI). Proportional hazards assumption was assessed for each variable in the model. For categorical variables, log(−log) survival plots were assessed for deviations from parallelism. Additionally, for both continuous and categorical variables, interaction terms were created with each variable and time, and the significance of each variable was tested. Absence of a significant interactive term was considered as meeting the proportional hazards assumption. The model was created using normalized HE4 levels and was adjusted for normalized age, sex, hypertension, hypercholesterolemia, diabetes mellitus, smoking status, congestive heart failure, and coronary artery disease without any interactive terms.

For the secondary objective, biomarker levels were compared between patients with AAA and controls, as well as in AAA patients who had suffered from a MACE versus those who had not, by the Mann–Whitney U Test. Simple linear regression was used to determine the association between biomarker levels at baseline and AAA diameter at baseline. Freedom from MACEs was determined by Kaplan–Meier curves, and differences between both groups (patients with a MACE versus no MACE) were compared by log-rank test.

Patients who did not complete the full 5-year follow-up period or were not available for telephone follow-up were considered censored at the time of their last follow-up contact. These patients were included in both the Kaplan–Meier survival analysis and the Cox proportional hazards regression model as censored observations. Statistical significance was set as *p* < 0.05. All analyses were conducted using SPSS Version 29.0.0.0 (241) and GraphPad Prism Version 10.1.0 (264).

## 3. Results

### 3.1. Patient Demographics and Clinical Characteristics

Overall, 452 patients were recruited for this study, of which 343 had AAA with an aneurysm ≥ 3 cm, and 109 were controls with abdominal aortas measuring < 3 cm. Patients with AAA had an average maximum aneurysm diameter of 4.3 ± 0.9 cm and control patients without AAA had an average maximum aortic diameter of 2.2 ± 0.5 cm (*p* < 0.001). Patients with AAA were significantly older (74 ± 9 vs. 67 ± 11 years, *p* < 0.001), more likely to be male (84% vs. 65%, *p* < 0.001), past smokers (55% vs. 37%, *p* < 0.001), taking cholesterol-lowering medications (79% vs. 71%, *p* = 0.024), and less likely to be taking insulin (3% vs. 7%, *p* = 0.047) (Table 1).

### 3.2. Plasma Protein Concentrations in Patients with AAA Versus Controls

**Figure A1:**
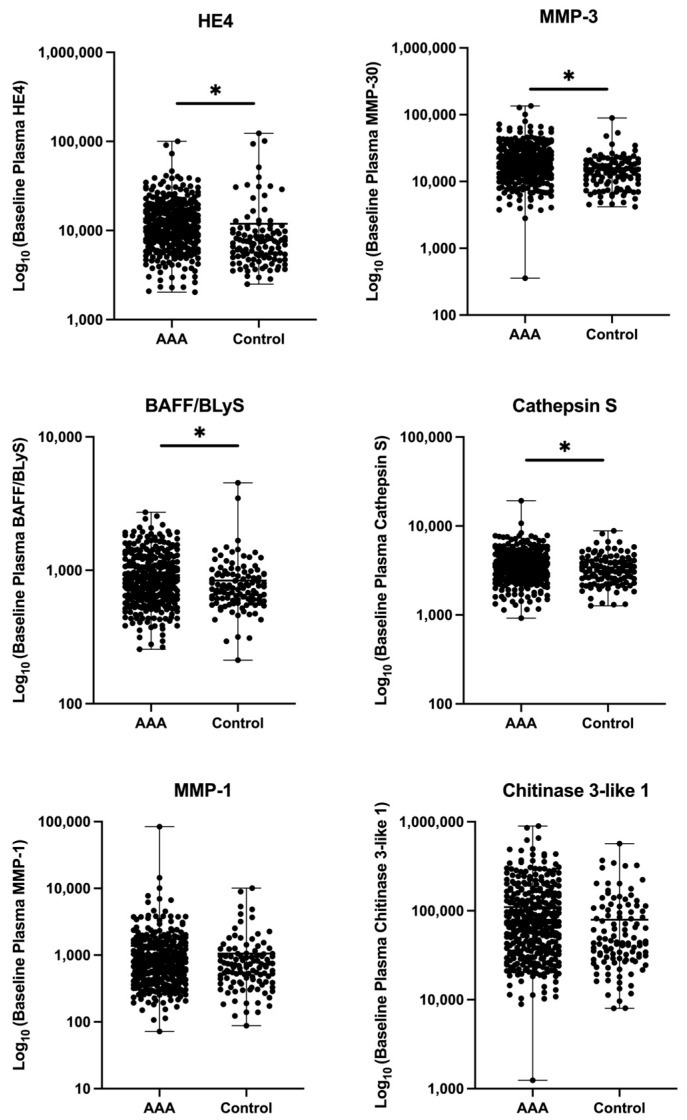
Comparison of baseline plasma biomarker levels between patients with abdominal aortic aneurysm (AAA) and controls. Scatters show the distribution of six plasma biomarkers: Human Epididymis Protein 4 (HE4), Matrix Metalloproteinase-3 (MMP-3), B-cell Activating Factor (BAFF/BLyS), Cathepsin S, Matrix Metalloproteinase-1 (MMP-1), and Chitinase 3-like 1. Each dot represents an individual participant. Y-axes represent biomarker concentrations on a log_10_ scale (pg/mL). Asterisks (*) represent statistically significant differences between groups (*p* < 0.05).

Patients with AAA had elevated levels of all six biomarkers of interest compared to controls; however, only four were significantly elevated in the AAA group compared to controls. Specifically, patients with AAA had higher plasma levels of HE4 (13.594 ± 10.702 vs. 11.889 ± 17.952 ng/mL, *p* < 0.0001), MMP-3 (22.138 ± 15.794 vs. 15.894 ± 10.879 ng/mL *p* < 0.0001), BAFF/BLyS (921.7 ± 421.3 vs. 838.6 ± 518.1 pg/mL, *p* = 0.0014), and Cathepsin S (3.728 ± 1.683 vs. 3.424 ± 1.388 ng/mL, *p* = 0.0156) (Table 2). Scatter plots can be found in the Appendix A (Figure A1).

### 3.3. Relationship Between Biomarkers and Abdominal Aortic Diameter at Baseline

To determine whether the biomarkers were related to AAA and aortic diameter, simple linear regression was conducted to determine the associations between normalized biomarker levels at baseline and aortic diameter at baseline. The coefficients for each biomarker are presented in Table 3. Of the six biomarkers, three biomarkers had a statistically significant positive associations with abdominal aortic diameter, suggesting that increases in these biomarkers were associated with increasing abdominal aortic diameter. With every normalized unit increase in HE4/WFDC2, MMP-3, and Chitinase 3-like 1, there was an increase in abdominal aortic diameter by 0.154 (95% CI: 0.032–0.276, *p* = 0.013), 0.186 (95% CI: 0.064–0.309, *p* = 0.003), and 0.231 (0.110–0.353, *p* < 0.001) centimeters, respectively.

### 3.4. Associations Between Biomarkers and Major Adverse Cardiovascular Events

All patients were followed over a period of 5 years (60 months) with an average follow-up time of 59 ± 2 months. Patients with AAA had significantly higher rates of MACEs compared to controls (76 events vs. 13, *p* = 0.019), as shown in Table 4.

All patients were then split into patients who suffered from a MACE versus those who did not suffer from a MACE within the 5-year period, and biomarker levels were then compared between the two groups. Patients with MACEs had significantly elevated levels of HE4 (19.123 ± 21.889 vs. 11.727 ± 8.818, *p* < 0.001) and MMP-1 (1338 ± 4585 vs. 1142 ± 971.3, *p* = 0.013) (Table 5). Comparisons between demographic and clinical characteristics can be found in Appendix A (Table A1).

To determine whether HE4 and MMP-1 were associated with an increased risk of MACEs, Cox regression analysis was conducted. All variables included in the model met the proportional hazard assumption. HE4 was a significant positive predictor of both MI (HR 1.403, 95% CIL 1.186–1.66, *p* <0.001) and MACEs (HR 1.353, 95% CI: 1.147–1.596, *p* < 0.001), even after adjusting for age, sex, hypertension, hypercholesterolemia, diabetes mellitus, smoking status, congestive heart failure, and coronary artery disease. MMP-1, however, was not significantly associated with any outcome (Table 6). The only other variable that was significantly associated with MACEs was coronary artery disease, with a HR of 2.373 (95% CI 1.502–3.749, *p* < 0.001) (Table A2).

### 3.5. Risk Stratification for MACEs Based on HE4 Levels

**Figure 1:**
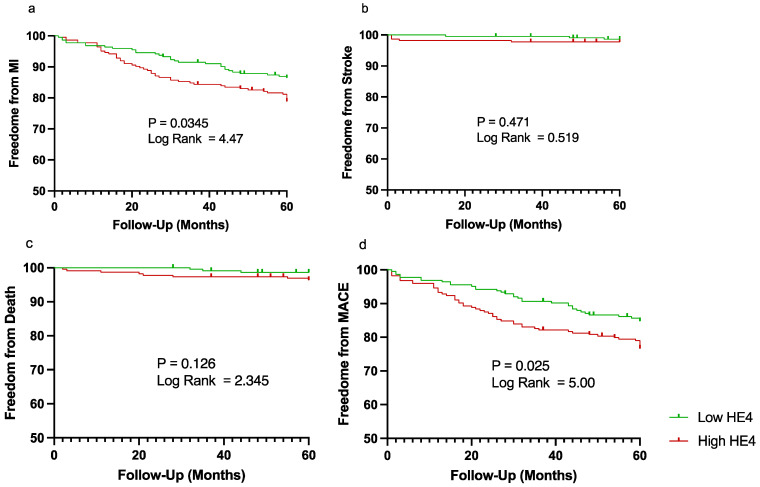
Kaplan–Meier curves demonstrating freedom from major adverse cardiovascular events (MACEs) over a period of 5 years (60 months). Patient were split into low (shown in green) vs. high (shown in red) human epididymis protein 4 (HE4) based on median plasma level for (**a**) myocardial infarction (MI), (**b**) stroke, (**c**) cardiovascular-related death, and (**d**) MACEs. Vertical lines represent censored patients.

All patients were split into low or high HE4 levels based on median HE4 level (9.338 ng/mL). Those with higher HE4 were more likely to have AAA, were significantly older, had higher levels of hypertension, congestive heart failure, and coronary artery disease, and were more likely to be taking angiotensin-converting enzyme inhibitors/angiotensin receptor blockers (ACE/ARBi) (Table 7). Based on Kaplan–Meier analysis, patients with high HE4 levels had significantly lower freedom from MI (86.47% vs. 78.91%, *p* = 0.035) and freedom from MACEs (84.80% vs. 76.67%, *p* = 0.025) after the 5-year period compared to patients with low HE4 levels (Figure 1).

Patients within the high-HE4 group also had significantly higher rates of MI (22% vs. 14%, *p* = 0.039) and MACEs (24% vs. 15%) when compared to those with low HE4 levels. There was no significant difference between the two groups in the rates of stroke and cardiovascular-related death (Table 8).

### 3.6. Association Between HE4 and MACEs in Patients with AAA

To determine the association between HE4 and MACEs specifically in patients with AAA, AAA patients were split into those who had suffered from a MACE (*n* = 76) and those who had not suffered from a MACE (*n* = 267). Patients with AAA who had experienced a MACE had significantly elevated levels of HE4 compared to patients with AAA who had not experienced a MACE (15,955 ± 14,875 vs. 12,922 ± 9101, respectively, *p* = 0.029) (Table 9). Demographic and clinical characteristics can be found in Appendix A (Table A3).

HE4 levels in patients with AAA were significantly associated with both MI and MACEs. Increasing levels of HE4 were associated with an increased risk of MI (HR 1.275, 95% CI: 1.070–1.518, *p* = 0.006) and MACEs (HR 1.249, 95% CI: 1.057–1.476, *p* = 0.009) when adjusted for age, sex, hypertension, hypercholesterolemia, diabetes mellitus, smoking status, congestive heart failure, and coronary artery disease. Increasing levels of HE4 were significantly associated with cardiovascular death, but this did not remain significant after adjusting for age, sex, hypertension, hypercholesterolemia, diabetes mellitus, smoking status, congestive heart failure, and coronary artery disease (Table 10). In the regression analysis, coronary artery disease was also significantly associated with MACEs when adjusting for other covariates (Appendix A, Table A4).

## 4. Discussion

In this study, the results may indicate that HE4 could act as a meaningful prognostic biomarker for predicting MACE. Specifically, with every one unit increase in normalized HE4, there was a 35.3% increased risk of MACEs within five years after adjusting for age, sex, hypertension, hypercholesterolemia, diabetes, smoking status, congestive heart failure, and coronary artery disease. When looking specifically at patients with AAA, HE4 was still significantly associated with MACEs after adjusting for confounders, demonstrating that with every one unit increase in normalized HE4, there is an increased risk of MACEs by 24.9%. We also demonstrated that four biomarkers were significantly elevated in patients with AAA (HE4/WFDC2, MMP-3, BAFF/BlyS, and Cathepsin S), and three were linearly associated with AAA diameter at baseline (HE4/WFDC2, MMP-3, Chitinase-3 like 1).

AAA is a silent disease with life-threatening consequences [27]. Patients with stable AAA without aortic repair have a significantly elevated risk of cardiovascular disease (hazard ratio (HR) 1.672, 95% confidence interval (CI): 1.522–1.835) and MACEs, including myocardial infarction (MI, HR 1.7, 95% CI: 1.479–1.953), cerebrovascular attack (CVA, HR 1.629, 95% CI: 1.443–1.839), and all-cause mortality (HR 2.544, 95% CI: 2.377–2.722) when compared to matched controls without AAA [28]. Hence, early detection of patients with elevated risk of MACEs at baseline is necessary to streamline treatment and surgical planning [11].

Currently, no strategies exist to stop the progression of aneurysm growth; however, early management can help slow the growth rate [29]. Once the aneurysm has reached ≥5.5 cm, it is recommended that surgical treatment is provided, as the risk of aortic rupture is approximately 9–10% per year [13]. Two strategies are currently available for surgical management: open abdominal aortic repair, where a large incision is made within the abdomen to access the aorta, or endovascular aortic repair (EVAR), where a smaller incision is created, often in the femoral artery in the groin, and a catheter is used to place a stent-graft within the diseased region [13]. Open surgical repair has a longer recovery period and higher risk of post-operative adverse events, including ischemia, MI, stroke, sexual dysfunction, and claudication [30,31]. EVAR is known to have lower 30-day mortality, lower blood loss, and shorter recovery times; however, the disadvantages of EVAR include increased rates of stent complications such as endoleaks, endograft migration, collapse, kinking, stenosis, and the need for re-intervention [32]. Currently, there is a lack of prognostic biomarkers available to stratify patients with AAA at baseline who are at an increased risk of cardiovascular complications. Biomarkers that predict MACEs can help stratify patients and allow physicians to initiate more intensive medical treatment to prevent AAA growth and reduce adverse outcomes and the need for surgical intervention. A study in 2019 determined that patients who had higher risk factors for cardiac disease had significantly lower rates of MI and cardiovascular death with EVAR compared to open repair [33]. When those patients with higher risk reach the cut-off of aneurysmal size for surgical intervention, physicians can make more informed decisions about which surgical intervention may be the best fit.

The Society for Vascular Surgery guidelines currently provide the Vascular Study Group of New England (VSGNE) risk prediction model as a method to stratify patients undergoing elective AAA repair into mortality risk categories based on clinical and procedural factors [13,34]. Notably, this model lacks a plasma protein biomarker that may reflect underlying pathophysiological mechanisms of the disease. Our findings suggest that HE4 has a prognostic value in predicting MACEs, and future research could determine whether adding such a marker to these clinically accepted risk stratification models improves their predictive capabilities. Currently, machine learning models have also become increasingly effective in predicting outcomes in clinical settings due to their ability to determine more complex and non-linear relationships between characteristics of interest [35]. Investigating the use of machine learning models that integrate both clinical characteristics and HE4 may also lead to the discovery of more accurate risk prediction models that improve clinical decision making and patient outcomes.

Evidence suggests that HE4 is involved in the innate immune system and inflammation within epithelial membranes, increases fibrosis, and acts as a protease inhibitor [36,37,38]. It has also been shown to upregulate other proteinases, such as matrix metalloproteinases MMP-9, MMP-2, and cathepsin B, which have been implicated in AAA progression [39]. HE4 may contribute to increasing AAA diameter and MACEs through its involvement in these processes, exacerbating ECM breakdown and arterial wall weakening. Fibrosis is another critical pathophysiological process associated with AAA. The process involves the excessive remodeling of ECM components, leading to structural changes in the aortic wall. HE4 has been implicated in promoting fibrosis in various tissues [35]. In patients with AAA, increased HE4 levels may drive fibrotic remodeling, potentially leading to altered mechanical properties such as stiffening of the aortic wall and increased risk of not only aortic rupture but also cardiovascular changes and MACEs.

Previous studies have demonstrated a similar association between HE4 and cardiovascular events. A recent study also demonstrated that HE4 was associated with cardiovascular events in patients with chronic obstructive pulmonary disease (HR: 2.012; 95% CI, 1.773–4.469; *p* < 0.001) [40]. Another study demonstrated that HE4 was a predictor of ischemic cardiomyopathy (HR: 1.003, 95% CI: 1.001–1.005, *p* = 0.002) [41]. In this study, we have demonstrated the potential clinical association between HE4 and MACEs; however, its role in the progression of AAA remains unclear. HE4 has been studied as a potential marker of ovarian cancer; however, recent evidence suggests that it may also play a role within vascular pathophysiology [37,41]. In knockdown studies of HE4, silencing HE4 in cancer cells has led to a reduction in cell viability and invasiveness, which suggests a pro-survival and anti-apoptotic function [42]. Ischemia has been shown to upregulate HE4 expression through the hypoxia-inducible factor pathway; furthermore, HE4 can be upregulated by inflammatory cytokines such as interleukin-6 and tissue necrosis factor alpha [43,44]. In this study, elevated levels in AAA patients and its association with MACEs may be related to the compensatory survival mechanism or the inflammatory and fibrotic response to the vascular injury associated with AAA. Future research on in vivo models of vascular tissue would help to clarify whether HE4 is a marker of inflammation/fibrosis or an active contributor to vascular remodeling and cardiovascular events.

HE4 could play an important role in the management of AAA by potentially using its demonstrated prognostic capabilities to risk stratify patients and personalizing treatment plans to optimize outcomes and prevent MACEs. Patients exhibiting elevated HE4 levels could also be prioritized for more intensive surveillance and aggressive therapeutic interventions. For example, patients with elevated HE4 could benefit from aggressive cardiovascular risk factor control, such as intensified statin usage, strong antiplatelet therapy, and lifestyle modifications, including smoking cessation, if not already initiated, structured exercise therapies for weight loss, and diabetes management. Elevated HE4 levels may indicate a heightened inflammatory state and an increased risk of MACEs, suggesting that a less invasive approach such as EVAR might be preferable over open repair. HE4 may also potentially be a therapeutic target for the prevention of AAA progression and adverse cardiovascular events. The ability of HE4 to predict both MI and overall MACEs, as demonstrated in our study, highlights its potential as a promising prognostic biomarker in clinical practice. Further studies using HE4 as part of surgical decision-making would be required to determine its effectiveness in risk stratification and postoperative MACE reduction, as well as targeted therapy.

Focusing on the health system level, HE4 could be incorporated into screening in patients with AAA to identify patients who may benefit from more frequent monitoring or early cardiology referral. From a clinical perspective, the identification of HE4 as a potential prognostic marker can have a positive implication on the management of AAA. There is currently a lack of existing tools that predict long-term cardiovascular outcomes in this populations, and hence, incorporating HE4 into routine risk assessment can help stratify patients based on the likelihood of MACEs, thereby helping guide more intensive medical strategies and lifestyle interventions or influence the choice between endovascular or open repair. HE4 holds promise as a marker that can be clinically actionable and could complement current imaging and clinical risk assessment tools to help improve patient outcomes.

### Study Limitations

There are some limitations to our study that may limit the generalizability of our results. Firstly, this is a single-center study with a small sample size, which may not reflect the full patient population and may have led to a potentially reduced power for the outcomes of stroke and cardiovascular-related death. Also, some clinical characteristics, including BMI, race, and socioeconomic status, were not included in the analysis, which may further reduce the generalizability to the general population. HE4 has been associated with some other diseases, such as pulmonary fibrosis, ischemic cardiomyopathy, and pulmonary arterial hypertension; however, these were not included in our analysis [41,45]. Our study population was predominantly male, reflecting the known higher prevalence in males. Further studies of HE4 in a female population may be warranted. Secondly, patients were called over the telephone if a recent clinical follow-up was not available. Patients may not have accurately recalled whether events had occurred and hence this may have influenced the results. Also, due to the observational nature of this pilot prospective study, the possibility of residual confounding and selection bias cannot be entirely ruled out and may preclude causal inferences. Lastly, the degree of fibrosis was not quantified in patients with AAA, and due to the kinetic differences between biomarkers, the physiological mechanisms behind our observed results are difficult to determine. These results, however, are important and warrant further research on the use of HE4 as a prognostic biomarker for MACEs in patients with AAA.

## 5. Conclusions

In conclusion, our study has demonstrated that HE4 is associated with AAA diameter and can predict the risk of MACEs in 5 years in patients with AAA. However, given the single-center and observational nature of this study, multi-center validation in a diverse patient population is needed to validate our findings. Integration of a strong prognostic biomarker is necessary for risk stratification of patients with AAA, both to provide more rigorous medical therapy to reduce aortic diameter growth and for surgical treatment decision-making. Our study demonstrates that HE4 may be a contender to be used for the prognostication of AAA, allowing for better management and in turn reducing adverse cardiovascular events in this patient population; however, further research is needed before clinical application.

## Figures and Tables

**Table 1:** Clinical characteristics of patients with abdominal aoristic aneurysm (AAA, *n* = 343) and controls (*n* = 109). Continuous variables are presented as mean ± standard deviation (SD), and categorical variables are presented as number of patients (percentage) (N(%)). Comparisons between AAA and controls with *p*-value < 0.05 were considered statistically significant and are bolded. ACE/ARBi, angiotensin-converting enzyme inhibitor/angiotensin receptor blocker.

	Overall (*n* = 452)	AAA (*n* = 343)	Control (*n* = 109)	*p*-Value
	Mean ± SD			
**Age (Years)**	**72 ± 10**	**74 ± 9**	**67 ± 11**	**<0.001**
**Aneurysm Size (cm)**	**3.7 ± 1.2**	**4.3 ± 0.9**	**2.2 ± 0.5**	**<0.001**
	*n* (%)			
**Sex (Male)**	**362 (80)**	**291 (84)**	**71 (65)**	**<0.001**
Hypertension	312 (69)	239 (70)	73 (67)	0.635
Hypercholesterolemia	349 (77)	269 (78)	80 (73)	0.295
Diabetes Mellitus	119 (26)	85 (25)	34 (31)	0.212
Smoking Status				
Current	119 (26)	95 (28)	24 (22)	0.263
**Past**	**230 (50)**	**190 (55)**	**40 (37)**	**<0.001**
**Never**	**103 (23)**	**58 (17)**	**45 (41)**	**<0.001**
Congestive Heart Failure	17 (4)	13 (4)	4 (4)	0.999
Coronary Artery Disease	153 (34)	123 (36)	30 (28)	0.131
Medication				
**Statin**	**348 (77)**	**271 (79)**	**77 (71)**	**0.027**
ACE/ARBi	217 (48)	168 (49)	49 (45)	0.651
Beta Blocker	162 (36)	122 (36)	40 (37)	0.727
Calcium Channel Blocker	108 (24)	85 (25)	23 (21)	0.517
Oral Antihyperglycemic	81 (18)	58 (17)	23 (21)	0.245
**Insulin**	**18 (4)**	**10 (3)**	**8 (7)**	**0.047**
Aspirin	221 (49)	173 (50)	48 (44)	0.078

**Table 2:** Biomarker levels between patients with abdominal aoristic aneurysm (AAA, *n* = 343) and controls (*n* = 109). Comparisons between AAA and controls with *p*-value < 0.05 were considered statistically significant. Human epididymis protein 4, HE4; Matrix metalloproteinase, MMP; B-cell activating factor, BAFF/BLyS.

	Control	AAA	*p*-Value
HE4/WFDC2 (ng/mL)	11.889 ± 17.952	13.594 ± 10.702	<0.001
MMP-3 (ng/mL)	15.894 ± 10.879	22.138 ± 15.794	<0.001
BAFF/BLyS (pg/mL)	838.6 ± 518.1	921.7 ± 421.3	0.001
Cathepsin S (ng/mL)	3.424 ± 1.388	3.728 ± 1.683	0.016
MMP-1 (ng/mL)	1.056 ± 1.466	1.377 ± 4.669	0.067
Chitinase 3-like 1 (ng/mL)	79.379 ± 88.359	109.729 ± 117.956	0.064

**Table 3:** Simple linear regression to determine the association between biomarker levels and abdominal aortic diameter. Human epididymis protein 4, HE4; Matrix metalloproteinase, MMP; B-cell activating factor, BAFF/BLyS. Coefficient (β) represents the value of each centimeter increase in aortic diameter with every 1 unit increase in z-score-normalized biomarker level.

Biomarker	Coefficient (β)	95% Confidence Interval	*p*-Value
HE4/WFDC2	0.154	0.032–0.276	0.013
MMP-3	0.186	0.064–0.309	0.003
BAFF/BLyS	0.108	−0.016–0.231	0.231
Cathepsin S	0.045	−0.79–0.168	0.480
MMP-1	0.081	−0.043–0.205	0.199
Chitinase 3-like 1	0.231	0.110–0.353	<0.001

**Table 4:** Major adverse cardiovascular events (MACEs) in patients with and without abdominal aortic aneurysm (AAA) after 5-year follow-up. Comparisons between AAA and controls with *p*-value < 0.05 were considered statistically significant and are bolded.

	Overall (*n* = 452)	AAA (*n* = 343)	Control (*n* = 109)	*p*-Value
Myocardial Infarction	81 (17)	68 (20)	13 (12)	0.063
Stroke	8 (2)	8 (2)	0 (0)	0.208
Cardiovascular-Related Death	11 (2)	9 (3)	2 (2)	>0.999
**MACEs**	**89 (20)**	**76 (22)**	**13 (12)**	**0.019**

**Table 5:** Biomarker levels between patients who suffered from a major adverse cardiovascular event (MACE, *n* = 89) and those that did not (No MACE, *n* = 363). Comparisons of MACE and No MACE with *p*-value < 0.05 were considered statistically significant and are bolded. Human epididymis protein 4, HE4; Matrix metalloproteinase, MMP; B-cell activating factor, BAFF.

	MACE (*n* = 89)	No MACE (*n* = 363)	*p*-Value
**HE4/WFDC2 (ng/mL)**	**19.123 ± 21.889**	**11.727 ± 8.818**	**<0.001**
MMP3 (ng/mL)	21.814 ± 13.609	20.342 ± 15.311	0.150
BAFFBLyS (pg/mL)	940.8 ± 401.4	892.1 ± 458	0.105
Cathepsin S (ng/mL)	3.72 ± 1.438	3.639 ± 1.664	0.391
**MMP1 (pg/mL)**	**1338 ± 4585**	**1142 ± 971.3**	**0.013**
Chitinase-3 like 1 (ng/mL)	99.297 ± 122.606	103.173 ± 109.67	0.855

**Table 6:** Multivariate Cox proportional hazard regression models for associations between every one unit increase in z-score-normalized levels of (A) HE4 and (B) MMP-1 and major adverse cardiovascular events (MACEs). Analysis was adjusted for age, sex, hypertension, hypercholesterolemia, diabetes, smoking status, congestive heart failure, and coronary artery disease. Human epididymis protein 4, HE4; Matrix metalloproteinase, MMP; hazard ratio, HR.

HE4	Unadjusted HR (95% CI)	*p*-Value	Adjusted HR (95% CI)	*p*-Value
**Myocardial Infarction**	**1.335 (1.196–1.491)**	**<0.001**	**1.403 (1.186–1.66)**	**<0.001**
Stroke	0.843 (0.326–2.181)	0.724	0.552 (0.144–2.113)	0.385
Cardiovascular-Related Death	1.313 (0.980–1.760)	0.068	1.233 (0.740–2.056)	0.421
**MACEs**	**1.311 (1.175–1.462)**	**<0.001**	**1.353 (1.147–1.596)**	**<0.001**
**MMP-1**	**Unadjusted HR (95% CI)**	***p*-Value**	**Adjusted HR (95% CI)**	***p*-Value**
Myocardial Infarction	0.904 (0.568–1.438)	0.67	0.835 (0.440–1.584)	0.581
Stroke	1.056 (0.695–1.603)	0.799	1.043 (0.688–1.581)	0.844
Cardiovascular-Related Death	0.881 (0.205–3.785)	0.865	0.878 (0.135–5.536)	0.878
MACEs	0.946 (0.688–1.299)	0.730	0.913 (0.604–1.381)	0.667

**Table 7:** Clinical characteristics of patients with low HE4 levels (*n* = 226) vs. high HE4 levels (*n* = 226) based on median HE4 levels (9.338 ng/mL). Continuous variables are presented as mean ± standard deviation (SD), and categorical variables are presented as number of patients (percentage) (N(%)). Comparisons between low HE4 levels vs. high HE4 levels with *p*-value < 0.05 were considered statistically significant and are bolded. ACE/ARBi, angiotensin-converting enzyme inhibitor/angiotensin receptor blocker.

	Low HE4 (226)	High HE4 (226)	*p*-Value
	Mean ± SD		
**Age**	**68.87 + 10.13**	**76.04 + 8.101**	**<0.001**
	*n* (%)		
**AAA**	**147**	**196**	**<0.001**
Sex (Male)	180	182	0.999
**Hypertension**	**144**	**168**	**0.025**
Hypercholesterolemia	170	179	0.433
Diabetes Mellitus	56	63	0.522
Smoking Status			
Current	56	63	0.455
Past	108	122	0.188
Never	62	41	0.019
**Congestive Heart Failure**	**4**	**13**	**0.045**
**Coronary Artery Disease**	**62**	**91**	**0.005**
Medication			
Statin	165	181	0.231
**ACE/ARBi**	**95**	**120**	**0.043**
Beta Blocker	71	90	0.111
Calcium Channel Blocker	48	59	0.317
Oral Antihyperglycemic	38	43	0.712
Insulin	7	11	0.473
Aspirin	101	120	0.188

**Table 8:** Major adverse cardiovascular events (MACEs) in patients split into two groups based on high human epididymis protein 4 (HE4) vs. low HE4 levels by median HE4 level (9.338 ng/mL). Comparisons between the high-HE4 vs. low-HE4 group with *p*-value < 0.05 were considered statistically significant and are bolded.

	Overall (*n* = 452)	High HE4 (*n* = 226)	Low HE4 (*n* = 226)	*p*-Value
**Myocardial Infarction**	**81 (17)**	**49 (22)**	**32 (14)**	**0.039**
Stroke	8 (2)	5 (2)	3 (1)	0.476
Cardiovascular-Related Death	11 (2)	8 (4)	3 (1)	0.127
**MACEs**	**89 (20)**	**54 (24)**	**35 (15)**	**0.025**

**Table 9:** Biomarker levels between patients who suffered from a major adverse cardiovascular event (MACE, *n* = 76) and those that did not (No MACE, *n* = 267) in patients with abdominal aortic aneurysm (AAA). Comparisons of MACE and No MACE with *p*-value < 0.05 were considered statistically significant. Human epididymis protein 4, HE4; Matrix metalloproteinase, MMP; B-cell activating factor, BAFF.

	AAA with MACE (*n* = 76)	AAA with No MACE (*n* = 267)	*p*-Value
HE4/WFDC2 (ng/mL)	15.955 ± 14.875	12.922 ± 9.101	0.029
MMP3 (ng/mL)	22.273 ± 16.395	21.661 ± 13.558	0.909
BAFF/BLyS (pg/mL)	910± 423	963 ± 417	0.177
Cathepsin S (ng/mL)	3.729 ± 1.749	3.724 ± 1.437	0.622
MMP1 (pg/mL)	1438 ± 5264	1164 ± 1035.274	0.111
Chitinase-3 like 1 (ng/mL)	112.163 ± 11.4204	101.178 ± 130.743	0.383

**Table 10:** Multivariate Cox proportional hazard regression models for associations between every one unit increase in z-score-normalized HE4 and major adverse cardiovascular events in patents with AAA. Analysis was adjusted for age, sex, hypertension, hypercholesterolemia, diabetes, smoking status, congestive heart failure, and coronary artery disease. Abdominal aortic aneurysm, AAA; human epididymis protein 4, HE4; hazard ratio, HR; major adverse cardiovascular event, MACE.

	Unadjusted HR (95% CI)	*p*-Value	Adjusted HR (95% CI)	*p*-Value
Myocardial Infarction	1.241 (1.058–1.455)	0.008	1.275 (1.070–1.518)	0.006
Stroke	0.761 (0.283–2.044)	0.588	0.662 (0.207–2.124)	0.488
Cardiovascular-Related Death	1.373 (1.007–1.871)	0.045	1.212 (0.776–1.878)	0.404
MACE	1.207 (1.030–1.414)	0.020	1.249 (1.057–1.476)	0.009

## Data Availability

The raw data supporting the conclusions of this article will be made available by the authors on request.

## Abbreviations

The following abbreviations are used in this manuscript:

AAA	Abdominal Aortic Aneurysm
ACE/ARBi	Angiotensin-Converting Enzyme Inhibitor/Angiotensin Receptor Blocker
BAFF/BLyS	B-cell Activating Factor/B Lymphocyte Stimulator
CI	Confidence Interval
CVA	Cerebrovascular Attack (i.e., Stroke)
ECM	Extracellular Matrix
EVAR	Endovascular Aortic Repair
HE4/WFDC2	Human Epididymis Protein 4/Whey Acidic Protein Four-Disulfide Core Domain 2
HR	Hazard Ratio
MACE	Major Adverse Cardiovascular Events (Composite of MI, CVA, and Cardiovascular-Related Death)
MI	Myocardial Infarction
MMP-1/3/9	Matrix Metalloproteinase 1, 3, or 9
pg/mL	Picograms per Milliliter
SD	Standard Deviation
SVS	Society for Vascular Surgery

## Appendix A

**Table A1:** Clinical characteristics of patients who suffered from a major adverse cardiovascular event (MACE, *n* = 89) and those who did not (*n* = 363). Continuous variables are presented as mean ± standard deviation (SD) and categorical variables are presented as number of patients (percentage) (N(%)). Comparisons between MACE and No MACE with *p*-value < 0.05 were considered statistically significant and are bolded. ACE/ARBi: angiotensin-converting enzyme inhibitor/angiotensin receptor blocker.

	MACE (*n* = 363)	NO MACE (*n* = 89)	*p*-Value
	Mean ± SD		
Age (Years)	72 ± 10	74 ± 8	0.333
	*n* (%)		
Sex (Male)	291 (80)	71 (80)	>0.999
Hypertension	248 (68)	64 (72)	0.597
Hypercholesterolemia	279 (77)	70 (779	0.826
Diabetes Mellitus	88 (24)	31 (35)	0.058
Smoking Status			
Current	95 (26)	24 (27)	0.985
Past	183 (50)	47 (53)	0.774
Never	85 (23)	18 (20)	0.616
Congestive Heart Failure	15 (4)	2 (2)	0.598
**Coronary Artery Disease**	**108 (30)**	**45 (51)**	**<0.001**
Medication			
Statin	273 (75)	75 (84)	0.093
ACE/ARBi	171 (47)	46 (52)	0.512
Beta Blocker	124 (34)	38 (43)	0.167
Calcium Channel Blocker	88 (24)	20 (22)	0.832
Oral Antihyperglycemic	63 (17)	18 (20)	0.632
Insulin	11 (3)	7 (8)	0.074
Aspirin	171 (47)	50 (56)	0.157

**Table A2:** Hazard ratios (HRs) and 95% confidence intervals (95% CIs) for major adverse cardiovascular events (MACEs) in all patients (*n* = 452) for all variables included in multivariate Cox regression analysis. Reference categories were “Female” for sex, no medical history for clinical features, and “Never” for smoking status. Human Epididymis Protein 4 (HE4) levels and age were z-score-normalized for analysis.

	HR	95% CI		*p*-Value
		Lower	Upper	
**HE4**	**1.353**	**1.147**	**1.596**	**<0.001**
Age	1.001	0.978	1.025	0.925
Sex (Male)	0.970	0.568	1.655	0.911
Hypertension	0.827	0.504	1.358	0.452
Hypercholesterolemia	1.157	0.654	2.047	0.617
Diabetes Mellitus	1.408	0.881	2.248	0.153
Smoking Status				
Past	0.952	0.551	1.647	0.862
Current	1.036	0.554	1.937	0.913
Congestive Heart Failure	0.299	0.071	1.252	0.098
**Coronary Artery Disease**	**2.373**	**1.502**	**3.749**	**<0.001**

**Table A3:** Clinical characteristics of patients with AAA only who suffered from a major adverse cardiovascular event (MACE, *n* = 76) and those who did not (*n* = 267). Continuous variables are presented as mean ± standard deviation (SD) and categorical variables are presented as number of patients (percentage) (N(%)). Comparisons between MACE and No MACE with *p*-value < 0.05 were considered statistically significant and are bolded. ACE/ARBi: angiotensin-converting enzyme inhibitor/angiotensin receptor blocker.

	MACE (*n* = 267)	NO MACE (*n* = 76)	*p*-Value
	Mean ± SD		
Age (Years)	74 ± 8	74 ± 9	0.8053
	*n* (%)		
Sex (Male)	226 (85)	65 (86)	0.9937
Hypertension	186 (70)	53 (70)	>0.999
Dyslipidemia	207 (78)	62 (82)	0.5489
Diabetes Mellitus	62 (23)	23 (30)	0.2696
Smoking Status			
Current	74 (28)	21 (28)	>0.999
Past	148 (55)	42 (55)	>0.999
Never	45 (17)	13 (17)	>0.999
Congestive Heart Failure	11 (4)	2 (3)	0.7956
**Coronary Artery Disease**	**83 (31)**	**40 (53)**	**0.0009**
Medication			
**Statin**	**204 (76)**	**67 (88)**	**0.0394**
ACE/ARBi	128 (48)	40 (53)	0.554
Beta Blocker	91 (34)	31 (41)	0.3463
Calcium Channel Blocker	67 (25)	18 (24)	0.9199
Oral Antihyperglycemic	43 (16)	15 (20)	0.5674
Insulin	6 (2)	4 (5)	0.321
Aspirin	130 (49)	43 (57)	0.2785

**Table A4:** Hazard ratios (HRs) and 95% confidence intervals (95% CIs) for major adverse cardiovascular events (MACEs) in patients with AAA (*n* = 343) for all variables included in multivariate Cox regression analysis. Reference categories were “Female” for sex, no medical history for clinical features, and “Never” for smoking status. Human Epididymis Protein 4 (HE4) levels and age were z-score-normalized for analysis.

	HR	95% CI		*p*-Value
		Lower	Upper	
HE4	1.249	1.057	1.476	0.009
Age	0.825	0.648	1.051	0.119
Sex	0.993	0.518	1.904	0.984
Hypertension	0.760	0.453	1.276	0.300
Hypercholesterolemia	1.362	0.728	2.546	0.333
Diabetes Mellitus	1.263	0.743	2.148	0.388
Smoking Status				
Past	0.841	0.449	1.576	0.589
Current	0.712	0.349	1.452	0.350
Congestive Heart Failure	0.438	0.104	1.854	0.262
Coronary Artery Disease	2.493	1.534	4.052	<0.001
